# Socioeconomic Differences between Sexes in Surgically Treated Carpal Tunnel Syndrome and Ulnar Nerve Entrapment

**DOI:** 10.3390/epidemiologia3030027

**Published:** 2022-07-15

**Authors:** Malin Zimmerman, Ilka Anker, Erika Nyman

**Affiliations:** 1 Department of Translational Medicine—Hand Surgery, Lund University, 205 02 Malmö, Sweden; ilka.anker@med.lu.se; 2 Department of Orthopedic Surgery, Helsingborg Hospital, 252 23 Helsingborg, Sweden; 3 Department of Biomedical and Clinical Sciences, Linköping University, 581 83 Linköping, Sweden; erika.nyman@liu.se; 4 Department of Hand Surgery, Plastic Surgery and Burns, Linköping University Hospital, 581 85 Linköping, Sweden

**Keywords:** carpal tunnel syndrome, ulnar nerve entrapment, sex differences, surgical outcome, socioeconomic factors

## Abstract

We aimed to investigate socioeconomic differences between sexes and the influence on outcome following surgery for carpal tunnel syndrome (CTS) or ulnar nerve entrapment (UNE) at the elbow. Patients with CTS (*n* = 9000) or UNE (*n* = 1266) registered in the Swedish National Register for Hand Surgery (HAKIR) 2010–2016 were included and evaluated using QuickDASH 12 months postoperatively. Statistics Sweden (SCB) provided socioeconomic data. In women with CTS, being born outside Sweden, having received social assistance, and more sick leave days predicted worse outcomes. Higher earnings and the highest level of education predicted better outcomes. In men with CTS, more sick leave days and having received social assistance predicted worse outcomes. Higher earnings predicted better outcomes. For women with UNE, higher earnings predicted better outcomes. In men with UNE, only sick leave days predicted worse outcomes. In long-term follow up, socioeconomic status affects outcomes differently in women and men with CTS or UNE.

## 1. Introduction

Carpal tunnel syndrome (CTS) and ulnar nerve entrapment at the elbow (UNE) are the two most common peripheral nerve entrapments in the upper extremities. In the general population, CTS is 1.4 times more common in women than in men, but among older individuals, the prevalence in women is almost four times that in men [1,2]. Several studies have reported that women experience more discomfort and functional impairment before open carpal tunnel release than men, whereas satisfaction after surgery seems to be independent of sex [3,4,5,6]. Work-related risk factors for developing CTS include vibrating tools and repetitive work [7]. In CTS, lower socioeconomic status is related to more persisting disability following surgical treatment [8].

UNE is reported to be slightly more frequent in men than in women [9], and male sex is a risk factor for developing UNE [9,10]. Women report more symptoms both before and after surgery for UNE, and one study reported greater improvement in women than in men following surgery [11]. Additionally, smoking, lower education level, and heavy manual, stationary, and repetitive work have been associated with a greater risk of developing the disorder [12,13,14,15]. UNE is also frequent in professional drivers due to multiple occasions of minor pressure on the cubital tunnel [16,17]. In a previous study, we found that patients having surgery for UNE are generally socioeconomically deprived [18].

Hence, in both CTS and UNE, women rate their pre- and postoperative disability higher than men. We hypothesized that socioeconomic differences between the sexes may play a part in the noted differences in surgical outcome and aimed to (1) evaluate socioeconomic differences between women and men surgically treated for CTS and UNE and (2) study whether socioeconomic factors affect outcomes differently in women and men operated upon for CTS and UNE.

## 2. Materials and Methods

Data on patients 18 years and above operated on for CTS or UNE between 2010 and 2016 were identified in the Swedish National Register for Hand Surgery (HAKIR). Only surgeries due to primary CTS (ICD-10 diagnosis code G560 and KKÅ97 operation code ACC51) and UNE (ICD-10 diagnosis code G562 and KKÅ97 operation codes ACC53 (simple decompression), ACC43 (transposition), and NCK19 (medial epicondylectomy)) were included. To detect a difference between sexes regarding mean income, as a selected indicator of socioeconomic status (power of 80%, *p* = 0.05), an estimated sample size of approximately 100 patients was needed for CTS and 150 patients for UNE. To detect a difference in QuickDASH between sexes (power of 80%, *p* = 0.05), an estimation of approximately 130 patients with CTS and 100 patients with UNE were needed. Patients were accepted for inclusion when the diagnosis codes G560 and G562 were the primary or secondary but not the tertiary diagnosis. Patients were only included once (i.e., reoperations were not included, and if there was a reoperation registered in HAKIR during the study period, only the first surgery was included). The HAKIR register does not provide information on how a diagnosis is made. In Sweden, CTS diagnosis is supported by electrophysiological examination in approximately 70% of cases [19] and in UNE in approximately 80% of cases [20]; in the rest of the cases, diagnosis is based on patient history and clinical examination.

Patients provide informed consent prior to inclusion in the HAKIR register and are asked to fill out the Swedish version of the patient reported outcome measure (PROM) QuickDASH [21] preoperatively and at 3 and 12 months postoperatively. QuickDASH consists of eleven items on disability in the upper extremity, and a total score of 0–100 is calculated based on the patient’s answers, where 0 represents no disability and 100 represents the maximum possible disability. In the present study, we only analyzed QuickDASH 12 months postoperatively. Data on diabetes status were retrieved from the Swedish National Diabetes Register (NDR) through personal identifying numbers (originally as part of a different study on the same population; in the present study, they were only used in the regression analysis). The NDR is a nationwide quality register that includes patients aged 18 and above with diabetes [22]. The register provides information on a wide range of diabetes-related parameters, previously described in detail [19,23]. Each patient provides informed consent for inclusion in the register. The patient was considered diabetic if the diagnosis of diabetes was made before or in the same year as the diagnosis of CTS or UNE. Socioeconomic data were retrieved from Statistics Sweden (SCB), which collects data on the entire Swedish population. Patients with reused personal identification numbers (the most common reason is an immigrated person receiving the personal identification number from a deceased person) were excluded (*n* = 17; 14 in the CTS population and 3 in the UNE population).

Earnings were indexed to December 2016 and calculated as the mean earnings per year between the ages of 30 and 65 years to estimate earnings during working years. Sixty-five years was the most common retirement age in Sweden during the study period. A binned variable with a quarter of the patients in each group was created for the purpose of linear regression analysis. Data were available from 1990 to 2016. Sick days only included sick days >14 days in a row due to the way the social security system is designed in Sweden where the first fourteen days are paid by the employer. Hence, we had no data on sick leave <14 days, and the data set was focused on prolonged sick leave. Data were available from 1994 to 2016, and the mean sick leave was calculated as net days per employed year over 20 years of age. Social assistance is temporary financial support provided by the municipality in Sweden to an individual unable to financially support themselves or their family. Data on social assistance were individualized and are presented as the total amount received (SEK) during the years 1990–2016. Data on unemployment were available from 1992 to 2016 and are presented as mean days per year during the ages of 30–65 years. Education levels were classified as primary (9 years of compulsory school), upper secondary (9–12 years of education), and tertiary (>12 years of education).

### Statistics

Nominal data are presented as number (%). Normally distributed continuous data are presented as means (95% confidence interval). The independent samples *t*-test was used to investigate differences. Non-normally distributed data (QuickDASH scores) are presented as medians (interquartile ranges) and were compared using the Mann–Whitney U-test. Categorical variables were compared used the chi-square test. A multivariate linear regression analysis was used to investigate the effect of socioeconomic variables on the total QuickDASH score twelve months postoperatively. A *p*-value of <0.05 was considered statistically significant. All calculations were made in IBM SPSS Statistics version 27 for Mac (SPSS Inc., Chicago, IL, USA).

## 3. Results

During the study period, 9000 patients operated upon for CTS were included. In total, 1266 patients operated upon for UNE were included. UNE surgeries were 1128 simple decompressions, 137 transpositions, and one medial epicondylectomy.

### 3.1. Carpal Tunnel Syndrome

**Figure 1:**
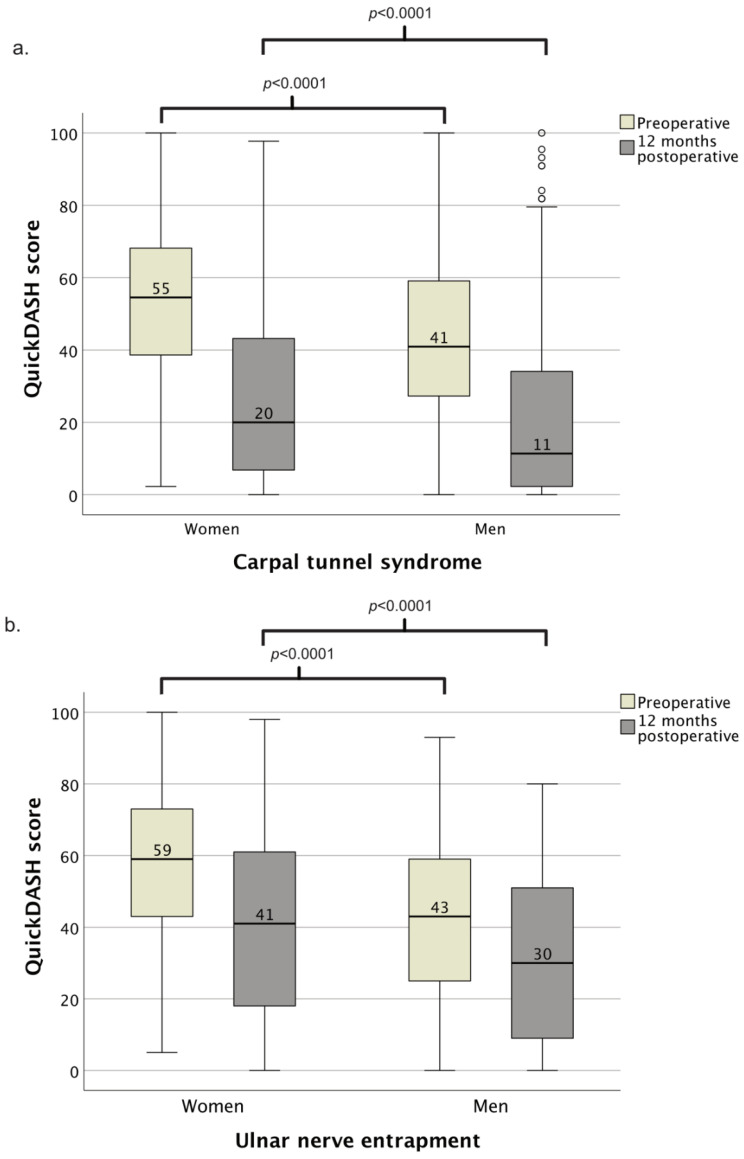
Boxplot of sex differences in QuickDASH scores for patients having surgery for carpal tunnel syndrome (**a**) or ulnar nerve entrapment at the elbow (**b**) preoperative and 12 months postoperative. QuickDASH–Quick Disabilities of Arm, Shoulder and Hand.

Women having surgery for CTS were younger than men having surgery for CTS (Table 1). More women than men with surgically treated CTS had higher education, were born outside of Sweden, and had received social assistance (Table 1). Women with CTS also earned less, less often had manual occupations, and had less unemployed days and more sick leave than men with CTS (Table 1). There were 3542 women (50%) and 2231 men (62%) who never had received any social assistance. In both men and women, manual workers had more sick leave than non-manual workers; female non-manual workers’ mean days/employed year was 19 (17–21) vs. female manual workers, 34 (31–36; *p* < 0.001); male non-manual workers, 12 (10–15) vs. male manual workers, 21 (19–23; *p* < 0.001). Preoperative QuickDASH scores were higher in women compared to men (Table 1). Twelve months postoperatively, the QuickDASH score was still higher in women than in men (Table 1, Figure 1). Women improved more in QuickDASH from the preoperative period to the 12-month postoperative period than men (median 27 (IQR 11–41), *n* = 590 vs. 20 (IQR 9–36), *n* = 269; *p* = 0.002).

In the regression analysis, age, being born outside Sweden, and more sick leave days were associated with higher QuickDASH scores at 12 months in women, and a high education level and more earnings predicted lower QuickDASH scores at 12 months (Table 2). In men with CTS, higher sick leave predicted higher QuickDASH scores at 12 months, whereas higher earnings predicted lower QuickDASH scores at 12 months (Table 2).

### 3.2. Ulnar Nerve Entrapment at the Elbow

Women having surgery for UNE were younger than men having surgery for UNE (Table 3). Women also earned less and less often had manual occupations and less unemployed days but had more social assistance and sick leave days than men with UNE (Table 3). There were 222 women (37%) and 296 men (45%) who never had received social assistance. In both men and women, manual workers had more sick leave than non-manual workers; female non-manual workers’ mean days/employed year was 30 (25–34) vs. female manual workers, 39 (33–45; *p* < 0.001); male non-manual workers, 20 (17–23) vs. male manual workers, 28 (24–31; *p* < 0.001). Preoperative QuickDASH scores were higher in women than in men (Table 3), both for decompressions and transpositions (*p* < 0.001). There were too few endoscopic releases and epicondylectomies to permit further analysis. Twelve months postoperatively, QuickDASH scores were still higher in women than in men (Table 3, Figure 1). Women improved more in QuickDASH from the preoperative period to the 12-month postoperative period than men (median 14 (IQR 5–26), *n* = 61 vs. 7 (−3–21), *n* = 54; *p* = 0.046).

**Figure 2:**
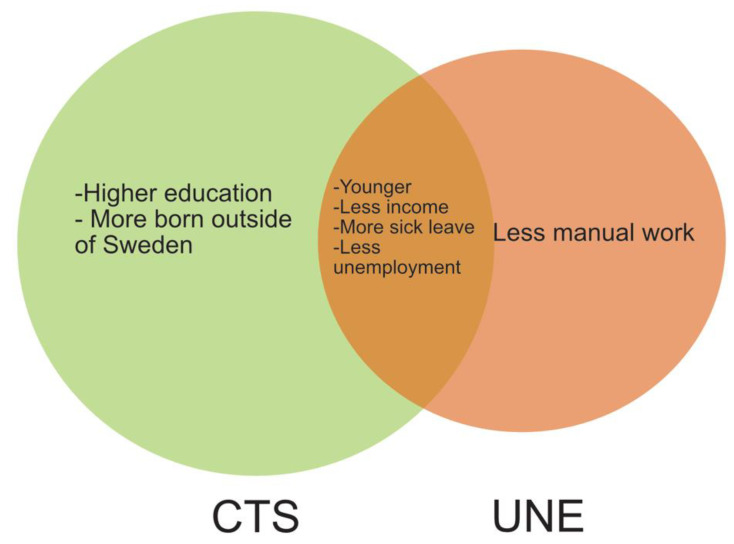
Schematic illustration (Venn diagram) showing characteristics of women surgically treated for carpal tunnel syndrome (CTS) or ulnar nerve entrapment at the elbow (UNE) in comparison to men (not shown)—differences and similarities in socioeconomic factors.

In the regression analysis, higher levels of earnings tended to predict lower QuickDASH scores at 12 months in women (Table 4). In men, more sick leave days predicted higher QuickDASH scores at 12 months (Table 4). Differences and similarities are schematically presented in Figure 2.

## 4. Discussion

Our data show that among patients having surgery for either CTS or UNE, women are more socioeconomically deprived than men. Women in our population earned less, more often received social assistance, and had more sick days than men. Generally, socioeconomic factors affected outcomes differently in women and men, and was to a greater extent related to worse outcomes in patients with CTS.

The populations with CTS and UNE differed considerably in some respects. In patients having surgery for CTS, social assistance and longer sick leave predicted more postoperative disability in both sexes, and higher earnings were associated with better postoperative outcomes. In women having surgery for CTS, being born outside of Sweden predicted more postoperative disability, which was not seen in men. Higher education was an independent predictor of better postoperative outcomes in women, but not in men. In patients having surgery for UNE, socioeconomic factors were less associated with outcomes than in patients having surgery for CTS in both sexes. In women having surgery for UNE, the highest level of earnings predicted better outcomes, whereas in men, the only independent predictor was sick leave days, which was associated with more postoperative disability.

Current evidence suggests that sex differences in pain perception have a complex background [24]. Additionally, socioeconomic factors may have different impact on perceived health in women and men. In a Swedish study, lower education predicted worse perceived health and higher earnings predicted better perceived health in women, but not in men [25]. In general, pain syndromes are more common among women, and women with musculoskeletal disorders report higher at pain scores than men with the same disorders [26]. This is also true for women with CTS, and women score their disability higher with QuickDASH than men [3,4], even though men have more pronounced pathology on preoperative electrophysiological examinations [4,27]. This might be attributed to the higher prevalence of diabetes found among men [27], but it is possible that one should rely more on reported symptoms when evaluating women for open carpal tunnel release and more on neurography results when evaluating men. One large study concluded that female sex was prognostic for a better outcome 1–3 years after open carpal tunnel release [28], which is consistent with the reports of more nerve damage in men seen in electrophysiological examinations, and thus more limited room for improvement.

Padua et al. argued for a greater tolerance to carpal tunnel discomfort among men [4], and there might be a physiological explanation, since women seem to have lower nociceptive thresholds [29].

We also found that even though women having surgery for CTS were more educated and had less unemployment rates than men, they earned less, received more social assistance, and had more sick leave than men. There were also significantly more immigrants in the group of women having surgery for CTS than in the group of men having surgery for CTS. Being a woman born outside of Sweden also predicted worse QuickDASH results in the linear regression analysis. In the general Swedish population, according to official data, in 2016, more men than women received social assistance. Women were more educated than men [30] but also had more paid sick leave than men, which is in accordance with official data from the entire population. However, in our study, there were large differences regarding paid sick leave between women and men (18 days in CTS and 10 days in UNE), whereas in official data from the entire population in 2016, women had a mean of two more days than men [31]. We also found more unemployment in men, which seems to be the situation in the entire general population [32]. Women also generally have lower earnings than men, so our population seems to be comparable with the general population in this aspect [32].

Traditionally, men have been over-represented in heavy manual occupations, often with work tasks involving vibrating tools. In modern times, men still have more work that involves heavy lifting, while occupations requiring repetitive movements are equally common among the sexes [33]. Women, however, are over-represented in health care occupations [34]. Work in nursing homes, as a personal assistant, or as an assistance nurse at hospital wards often include heavy manual tasks when caring for patients, which might predispose one to musculoskeletal disease such as CTS and UNE. Manual work did not predict outcome at 12 months in our analysis when adjusting for all other socioeconomic factors. Patients who perform manual work tasks normally require longer sick leave before returning to work but may be as satisfied with surgery as patients who do not perform manual work tasks.

The main strength of this study was the large study population, and the main limitation was the response rate on the outcome questionnaire QuickDASH. However, the response rate was similar to response rates of other large registries [35,36], and the non-responders did not differ substantially from the responders in our material (data already published [37]). Another limitation is that the HAKIR register contains no clinical information, and hence, the only variable on concomitant disease that we could include was diabetes. We did not have data on smoking status, BMI, thyroid disease, rheumatoid arthritis, psychiatric disorders, or other possible factors that could affect the self-reported function and disability in patients having surgery for CTS or UNE.

## 5. Conclusions

There are certain socioeconomic differences between women and men surgically treated for peripheral nerve entrapments in the upper extremities. Socioeconomic status affects surgical outcomes differently in women and men with CTS or UNE at long-term follow up, which may be valuable information for the treating surgeon.

## Figures and Tables

**Table 1:** Socioeconomic factors in 9000 patients with surgically treated carpal tunnel syndrome.

	Women (*n* = 5988)	Men (*n* = 3012)	*p*-Value
Age (years; mean, 95% CI)	55 (55–56)	59 (58–60)	**<0.0001**
Diabetes at surgery (*n*, %)	715 (12)	544 (18)	**<0.0001**
Tertiary education (*n*, %)	2827 (47)	1164 (39)	**<0.0001**
Mean annual earnings, SEK 1000 (mean, 95% CI)	174 (171–177)	248 (243–254)	**<0.0001**
Born outside Sweden (*n*, %)	1108 (19)	403 (13)	**<0.0001**
Manual occupation (*n*, %)	2288 (38)	1203 (40)	**0.001**
Unemployed days (mean/year; mean, 95% CI)	12 (12–13)	14 (13–15)	**0.001**
Social assistance (total amount; SEK 100, mean, 95% CI)	212 (185–239)	173 (134–211)	**<0.001**
Sick leave (mean days/employed year; mean, 95% CI)	42 (38–45)	24 (22–26)	**<0.0001**
Preoperative QuickDASH score (median, IQR)	55 (39–68) *n* = 2342	43 (27–59) *n* = 1241	**<0.0001**
Postoperative QuickDASH score at 12 months (median, IQR)	20 (5–41) *n* = 1379	11 (2–34) *n* = 653	**<0.0001**

Independent samples *t*-test used for comparisons of means. Chi-square used to compare numbers. Statistically significant *p*-values (<0.05) marked as bold. Complete data on social assistance missing in 2745 patients. CI–Confidence Interval. IQR–Interquartile Range.

**Table 2:** Linear regression model for patients with surgically treated carpal tunnel syndrome.

	Women (*n* = 556)		Men (*n* = 270)	
	Unstandardized B-Coefficient (95% Confidence Interval)	*p*-Value	Unstandardized B-Coefficient (95% Confidence Interval)	*p*-Value
Age at surgery (years)	0.13 (−0.06–0.33)	0.17	−0.18 (−0.45–0.13)	0.25
Diabetes at surgery	3.4 (−2.0–8.7)	0.22	2.3 (−4.5–9.1)	0.51
**Migrant status**				
Born in Sweden (reference)				
Born outside Sweden	8.5 (3.1–13.8)	**0.002**	−0.01 (−9.1–9.0)	1.0
**Occupation**				
Nonmanual work (reference)				
Manual work	−1.6 (−6.2–3.0)	0.49	−0.3 (−7.8–7.1)	0.93
**Level of education**				
Low (reference)				
Middle	−4.9 (−10.3–0.3)	0.07	−3.4 (−9.8–2.9)	0.29
High	−10.5 (−16.2–−4.8)	**<0.001**	−0.75 (−8.2–6.7)	0.84
**Earnings (mean/year)**				
≤39,100 (reference)				
39,200–183,100	−6.9 (−13.1–−0.7)	**0.029**	−7.5 (−18.6–3.6)	0.18
183,200–280,700	−7.1 (−14.0–−0.2)	**0.045**	−13.3 (−24.7–−1.9)	**0.022**
>280,800	−10.3 (−18.5–−2.1)	**0.014**	−16.5 (−28.6–−4.4)	**0.008**
Social assistance (total amount, SEK 100)	0.007 (0.0–0.01)	0.052	0.008 (0.0–0.02)	0.056
Unemployment (mean days/year)	0.065 (−0.038–0.17)	0.22	−0.08 (−0.23–0.06)	0.26
Sick leave (mean days/year)	0.06 (0.02–0.1)	**0.003**	0.10 (0.02–0.18)	**0.019**

Dependent variable: total QuickDASH score at 12 months. Statistically significant *p*-values (<0.05) marked as bold.

**Table 3:** Socioeconomic factors in 1266 patients with surgically treated ulnar nerve entrapment.

	Women (*n* = 604)	Men (*n* = 662)	*p*-Value
Age (years; mean, 95% CI)	51 (40–52)	53 (52–54)	**0.004**
Diabetes at surgery (*n*, %)	57 (9)	91 (14)	**0.018**
Tertiary education (*n*, %)	161 (27)	146 (22)	0.066
Mean annual earnings, SEK 1000 (mean, 95% CI)	148 (138–159)	206 (197–220)	**<0.0001**
Born outside Sweden (*n*, %)	101 (17)	113 (17)	0.88
Manual occupation (*n*, %)	215 (36)	275 (42)	**0.033**
Unemployed days (mean/year; mean, 95% CI)	17 (15–19)	21 (18–23)	**0.027**
Social assistance (total amount, SEK 100, mean, 95% CI)	581 (406–756)	376 (264–488)	**0.021**
Sick leave (mean days/employed year, mean, 95% CI)	33 (30–36)	23 (21–26)	**<0.0001**
Preoperative QuickDASH score (median, IQR)	59 (43–73) (*n* = 199)	43 (25–59) (*n* = 227)	**<0.001**
Postoperative QuickDASH score at 12 months (median, IQR)	41 (18–61) (*n* = 161)	30 (9–52) (*n* = 140)	**0.002**

Independent samples *t*-test was used for comparisons of means and Mann–Whitney U-test for comparisons of medians. Chi-square was used to compare numbers. Statistically significant *p*-values (<0.05) marked as bold. Complete data on social assistance missing in 375 patients.

**Table 4:** Linear regression for patients with surgically treated ulnar nerve entrapment at the elbow.

	Women (*n* = 122)		Men (*n* = 110)	
	Unadjusted B- Coefficient (95% ConfidenceInterval)	*p*-Value	Unadjusted B- Coefficient (95% Confidence Interval)	*p*-Value
Age at surgery (years)	0.2 (−0.2–0.7)	0.34	0.60 (0.06–1.1)	**0.031**
Diabetes at surgery	−11.5 (−27.3–4.2)	0.15	8.4 (−2.8–19.7)	0.14
**Migrant status**				
Born in Sweden (reference)				
Born outside Sweden	−0.35 (−15.6–14.9)	0.15	−0.29 (−14.1–13.6)	0.97
**Occupation**				
Non-manual work (reference)				
Manual work	−8.9 (−19.1–1.4)	0.089	5.4 (−4.1–15.0)	0.26
**Level of education**				
Low (reference)				
Middle	−3.4 (−14.9–8.1)	0.57	2.7 (−9.1–14.6)	0.65
High	−9.3 (−22.7–4.2)	0.18	−4.5 (−17.8–8.7)	0.50
**Earnings (mean/year)**				
≤39,100 (reference)				
39,200–183,100	−10.9 (−26.5–4.6)	0.14	1.6 (−18.2–21.4)	0.87
183,200–280,700	−9.7 (−26.5–7.1)	0.25	7.0 (−12.5–26.5)	0.48
>280,800	−16.7 (−34.0- 0.7)	0.06	3.6 (−15.1–22.3)	0.70
Social assistance (total amount, SEK 100)	0.5 (−0.9–1.8)	0.49	1.0 (−0.52–2.6)	0.19
Unemployment (mean days/year)	0.01 (−0.27–0.29)	0.93	−0.02 (−0.2–0.2)	0.85
Sick leave (mean days/year)	0.14 (−0.02–0.29)	0.084	0.25 (0.09–0.4)	**0.003**

Dependent variable: total QuickDASH score at 12 months. Statistically significant *p*-values (<0.05) marked as bold.

## Data Availability

Relevant data are included within the paper. The complete and detailed individual data of all subjects cannot be publicly available for ethical and/or legal reasons as this would compromise patient privacy. The Regional and National Ethical Committee have imposed these restrictions. Data requests may be sent to the National Ethical Committee via the homepage of Etikprövningsmyndigheten in Sweden (etikprovningsmyndigheten.se (accessed on 12 January 2022)).
